# Enhancing the cytotoxicity of chemoradiation with radiation-guided delivery of anti-MGMT morpholino oligonucleotides in non-methylated solid tumors

**DOI:** 10.1038/cgt.2017.27

**Published:** 2017-07-28

**Authors:** P Ambady, Y J Wu, J M Walker, C Kersch, M A Pagel, R L Woltjer, R Fu, L L Muldoon, E A Neuwelt

**Affiliations:** 1 Department of Neurology, Oregon Health and Science University, Portland, OR, USA; 2 Veterans Affairs Medical Center, Portland, OR, USA; 3 Department of Radiation Medicine, Oregon Health and Science University, Portland, OR, USA; 4 Department of Pathology, Oregon Health and Science University, Portland, OR, USA; 5 School of Public Health, Department of Medical Informatics and Clinical Epidemiology, Oregon Health and Science University, Portland, OR, USA; 6 Department of Cell, Developmental and Cancer Biology, Oregon Health and Science University, Portland, OR, USA; 7 Department of Neurosurgery, Oregon Health and Science University, Portland, OR, USA; 8 Office of Research and Development, Department of Veterans Affairs Medical Center, Portland, Oregon, USA

## Abstract

The DNA repair enzyme O^6^-methylguanine DNA methyltransferase (MGMT) is epigenetically silenced in some tumors by MGMT gene promoter methylation. MGMT-hypermethylated solid tumors have enhanced susceptibility to the cytotoxic effects of alkylating chemotherapy such as temozolomide, compared with non-methylated tumors. In glioblastoma, subjects with MGMT hypermethylation have significantly longer survival rates after chemoradiotherapy. We report the first successful use of a non-ablative dose of ionizing radiation to prime human cancer cells to enhance the uptake of unmodified anti-MGMT morpholino oligonucleotide (AMON) sequences. We demonstrate >40% reduction in the *in vitro* proliferation index and cell viability in radiation-primed MGMT-expressing human solid tumor cells treated with a single dose of AMONs and temozolomide. We further demonstrate the feasibility of using a non-ablative dose of radiation *in vivo* to guide and enhance the delivery of intravenously administered AMONs to achieve 50% MGMT knockdown only at radiation-primed tumor sites in a subcutaneous tumor model. Local upregulation of physiological endocytosis after radiation may have a role in radiation-guided uptake of AMONs. This approach holds direct translational significance in glioblastoma and brain metastases where radiation is part of the standard of care; our approach to silence MGMT could overcome the significant problem of MGMT-mediated chemoresistance.

## Introduction

Glioblastoma is an aggressive primary brain tumor that carries a poor prognosis even after aggressive resection and standard-of-care chemoradiotherapy (CRT). The standard regimen includes 60 Gy radiation delivered over 6 weeks in 1.8–2 Gy dose fractions per day concurrently with the oral alkylating chemotherapy temozolomide; this is followed by at least 6 additional months of adjuvant cycles of temozolomide.^1^ The cytotoxic effects of temozolomide in this setting are mediated by methylation of O^6^ and N^7^ positions of guanylic acid and the N^3^ position of adenine resulting in a continuous cycle of DNA base mismatch with eventual DNA strand breaks that ultimately triggers apoptosis. The DNA repair enzyme O^6^-methylguanine DNA methyltransferase (MGMT) is a suicide enzyme that unmethylates these sites to restore genome stability.^2, 3, 4, 5, 6, 7, 8^ Via this mechanism, MGMT enzyme expression has a key role in resistance to CRT.^2, 3, 5, 6^ The *MGMT* gene is epigenetically silenced in about 45% of glioblastoma cases by methylation of CpG islands, predominantly located in the 5′ gene promoter region.^9^ Tumor cells with MGMT gene promoter methylation have enhanced susceptibility to the cytotoxic effects of radiation and/or alkylating agents such as temozolomide due to their inability to repair the CRT-mediated DNA damage. This correlates with a significant survival benefit in glioblastoma patients with MGMT promoter methylation who receive CRT.^2, 8, 9, 10^ Silencing MGMT is thus a promising therapeutic target in glioblastoma and other solid tumors such as lung, breast and renal cell carcinoma where this enzyme is known to have a role in chemoresistance.^11, 12, 13^

Antisense morpholino oligonucleotides are short custom-built sequences of uncharged nucleic acid analogs that are composed of nitrogen base pairs bound to morpholino rings, instead of ribose or deoxyribose rings, linked through uncharged phosphorodiamidate groups instead of anionic phosphates.^14, 15^ They have emerged as excellent tools *in vitro* for blocking sites on RNA to prevent translation and thereby inhibit protein expression. We evaluated the use of novel anti-MGMT oligonucleotides (AMONs) developed on a neutral morpholino backbone that can effectively silence MGMT both *in vitro* and in tumor models.

The target specificity of morpholino oligomers and their stability *in vivo* makes them excellent candidates for potential therapeutic applications in humans.^14, 15, 16, 17^ However, intracellular delivery, especially into the brain across the blood–brain barrier (BBB), remains a major challenge and limits clinical translation for brain tumor therapy.^18, 19, 20^ Internalization of antisense oligomers is at least partially mediated by endocytosis.^21, 22, 23^ Ionizing radiation is known to enhance endocytosis.^24^ As RT is already an integral part of ‘standard-of-care’ treatment for primary and metastatic brain tumors, we proposed this as a method to potentially enhance cellular uptake of morpholinos.^1, 25^ In this report, we demonstrate the efficacy of a non-lethal dose of ionizing radiation to guide and enhance the intracellular uptake of unmodified antisense morpholino oligonucleotide sequences and to silence MGMT protein expression.

## Materials and methods

### Cell lines

Human T98G glioma, H460 and A549 non-small cell lung carcinoma (NSCLC) cell lines were obtained from American Type Culture Collection (ATCC; Rockville, MD, USA) and cultured in recommended culture medium supplemented with in 10% fetal bovine serum and 1% streptomycin/penicillin. Cells were maintained in an atmosphere containing 5% CO_2_ at 37 °C.

### Reagents

AMONs: Three AMON sequences and control sequences (Table 1) were synthesized and purchased from Gene Tools, LLC (Philomath, OR, USA). Temozolomide for injection was purchased Merck & Co., Inc. (Whitehouse Station, NJ, USA) and diluted in sterile water to a concentration of 2.5 mg ml^−1^. Mouse anti-tubulin antibody (T9026) was purchased from Sigma (St Louis, MO, USA). In western blotting studies, anti-MGMT (#2739), caspase 3 (#9662), caveolin1 (#3267), rab5 (#3547), clathrin (#4796), EEA1 (#3288), APPL1 (#3858) and phospho-p53 (Ser15) (#9284) antibodies were purchased from Cell Signaling Technology (Danvers, MA, USA). Mouse anti-dynamin 2 (sc-166526) and p27 (sc-527) antibodies were purchased from Santa Cruz Biotech. (Santa Cruz, CA, USA). For immunohistochemistry, Rabbit anti-MGMT (171195-1-AP) and ki67 (sc-15402) antibodies were purchased from Proteintech (Rosemont, IL, USA) and Santa Cruz Biotech, respectively.

### AMON delivery techniques

#### Scrape loading

Cell were placed on a flat surface and gently scraped off the surface with a sterile cell scraper (Sarstedt, Inc., Newton, NC, USA), according to the manufacturer’s protocol.^26^ Different doses or combinations of MGMT morpholinos were added; scraped cell suspension was then gently pipetted up and down twice and transferred to another culture plate.

#### Transfection agents

Cells were treated with transfection agent DharmaFECT (GE Dharmacon, Lafayette, CO, USA) and MGMT-morpholinos mixture (1:1; v/v) without scraping the cells according to the manufacturer’s protocol.^26^

#### Radiation-guided delivery

A total of 10^6^ cancer cells (T98G, H460 and A549) were exposed to different doses of radiation (0.5–12 Gy) at room temperature using a Philips gamma chamber (Philips Medical Systems, Andover, MA, USA). After irradiation, cells were treated with mixtures of MGMT morpholinos (#1:#2:#3=1:1:1; v/v/v) at different time points and at the final concentration of 15 μM. For the purpose of this study, non-ablative radiation is defined as radiation doses <8 Gy per fraction.^27^ Cells were radiated with different doses of radiation (0.5–12 Gy). Mixtures of AMONs (#1:#2:#3=1:1:1; 5 μM each) or no drug controls were added 1 day after radiation, and then temozolomide (150 μg ml^−1^, final concentration) was added into selected wells 2 days after AMONs. Cell viability was determined at specific time (1–4 days) after temozolomide treatment using WST-1 reagent (Abcam, Cambridge, MA, USA), following the manufacturer’s protocol.

### Cell viability assay and western blotting analyses

For western blotting analysis, cells (T98 G or H460 cell lines)) or tissues (H460 cells) were harvested and then washed with ice-cold phosphate-buffered saline (PBS) twice before the addition of RIPA lysis buffer containing protease and phosphatase inhibitors (Roche, Indianapolis, IN, USA). Protein concentration was quantified using the bicinchoninic acid protein assay. Equal amounts of protein (20 μg per lane) were loaded into each well and separated by 7.5 or 12% sodium dodecyl sulfate-polyacrylamide gel electrophoresis followed by transfer onto polyvinylidene difluoride membranes (Bio-Rad, Richmond, CA, USA). Membranes were blocked using 5% nonfat dry milk or 2% bovine serum albumin (in PBS with 0.05% tween-20. The blots were then incubated with primary antibodies (1:200–1:1000) overnight at 4 °C. Secondary antibody anti-rabbit or anti-mouse IgG conjugated to horse radish peroxidase (1:5000; Cell Signaling Technology) was incubated for 2 h at room temperature. Immunoblots were developed using the chemiluminescence detection system (Thermo Scientific, Waltham, MA, USA). Quantification of immunoblotting signal of respective proteins was performed using the UN-SCAN-IT Gel software (Silk Scientific, Inc., Orem, UT, USA).

### Immunohistochemistry

Cells were exposed to radiation (1 Gy) or control no radiation 1 day after plating; 24 h after radiation, cells were incubated with mixtures of AMONs (#1:#2:#3=1:1:1; 5 μM each); and temozolomide (150 μg ml^−1^, final concentration) was added into selected wells 2 days after AMONs. Cells were fixed in fresh 4% paraformaldehyde and permeabilized with 0.1% Triton X at room temperature for 15 min and then washed with PBS. Immunohistochemistry that was performed followed the procedure as described previously.^28^ Representative images are shown; all images are optical sections. All images were collected using identical settings on the microscope. MGMT was considered positive when uniform nuclear MGMT staining was detected, while immunostaining in the cytoplasmic and granular nuclear reactivity was considered negative as previously described.^29, 30^

### Confocal microscopy

T98G cells were exposed to radiation (1 Gy) or controls no radiation; either 24 h after radiation (1 Gy) or no radiation, cells were incubated with GFP-morpholino (1:500) and Texas red conjugated WGA (1:500) for 1 h at 37 °C and then fixed in fresh 3.7% paraformaldehyde at room temperature for 15 min and then washed with PBS. Membranes were not permeabilized. Images were collected the following day using an 60 × /1.4 NA oil immersion objective on an inverted Zeiss LSM780 confocal microscope maintained by the Oregon Health and Science University (OHSU) Advanced Light Microscopy Core (Carl Zeiss, Oberkochen, German); images were analyzed using FIJI/ImageJ (NIH, Bethesda, MD, USA, https://fiji.sc).^31^ Representative images are shown; all images are optical sections. All images were collected using identical settings on the microscope.

### Measurement of MGMT DNA methylation status

DNA from subcutaneous xenograft tumors (xenograft method described in detail below) was isolated using the DNeasy Blood & Tissue Kit from Qiagen International (Germantown, MD, USA). The percentage of methylation status of MGMT DNA at CpG islands in samples were measured at OHSU Knight Cancer Center Clinical Laboratories Service using pyrosequencing technique using Pyromark ID (Qiagen, Valencia, CA, USA).^32, 33^

### Subcutaneous tumor model and *in vivo* delivery of AMONs with non-ablative ionizing irradiation

The care and use of the animals was approved by the institutional animal care and use committee and was under the supervision of the Department of Comparative Medicine at OHSU. Female (200–250 g) nude (*rnu/rnu*) rats were selected from the breeding colony maintained in the animal facility at OHSU. For the subcutaneous tumor model, a total of 2.5 × 10^7^ cells were mixed (ratio 1:1; v/v) with Matrigel Basement Membrane Matrix (BD Biosciences, Bedford, MA, USA), and the mixture was injected subcutaneously into each hind limb. At day 7 after inoculation, the right subcutaneous tumor was exposed to single computer tomography-guided priming dose of radiation (5 Gy) using a Versa HD (Elekta, Stockholm, Sweden) linear accelerator in the Department of Radiation Oncology, and left subcutaneous tumor of the same animal without radiation served as control. One centimeter of tissue equivalent bolus material was overlaid onto the RT field to ensure adequate dose to the most superficial portion of the lesion. A field size of 3 × 5 cm^2^ was used and a half beam block technique was implemented in order to limit dose outside the RT field. Dose was confirmed using NanoDot (Landauer, Glenwood, IL, USA) optically stimulated luminescence dosimetry. *In vivo* animal experiments were performed in sets of two animals each where both received radiation to their right hind limb while only one animal received AMON, given intravenously (IV) as a total of 10.5 mg kg^−1^ mixtures of AMONs (#1:#2:#3=1:1:1) through the femoral vein 24 h after radiation. Tumor tissues were harvested 3 days after AMONs were administered and subjected to protein and DNA analysis. This experiment was repeated independently three times (*n*=6).

### Statistical analysis

The results were expressed as mean±s.d. For percentage of cells with nuclear MGMT staining or ki67+ staining, one-way analysis of variance models were used to compare differences between the different treatment groups. When there were two treatment factors (RT; AMON/TMZ), two-way analysis of variance models were used to compare relative p-p53 level, relative cell viability and relative MFMT level, where the relative values were obtained by dividing the raw data by the value from the control group. Multiple comparisons were adjusted using Tukey’s correction. Significance was determined at the 5% level, two sided. Statistical significance between treatment and control (or vehicle) groups or any two other groups was indicated by *P<0.05, **P<0.01 or *** *P*<0.001.

## Results

### Ionizing radiation enhances AMON delivery to silence MGMT

**Figure 1:**
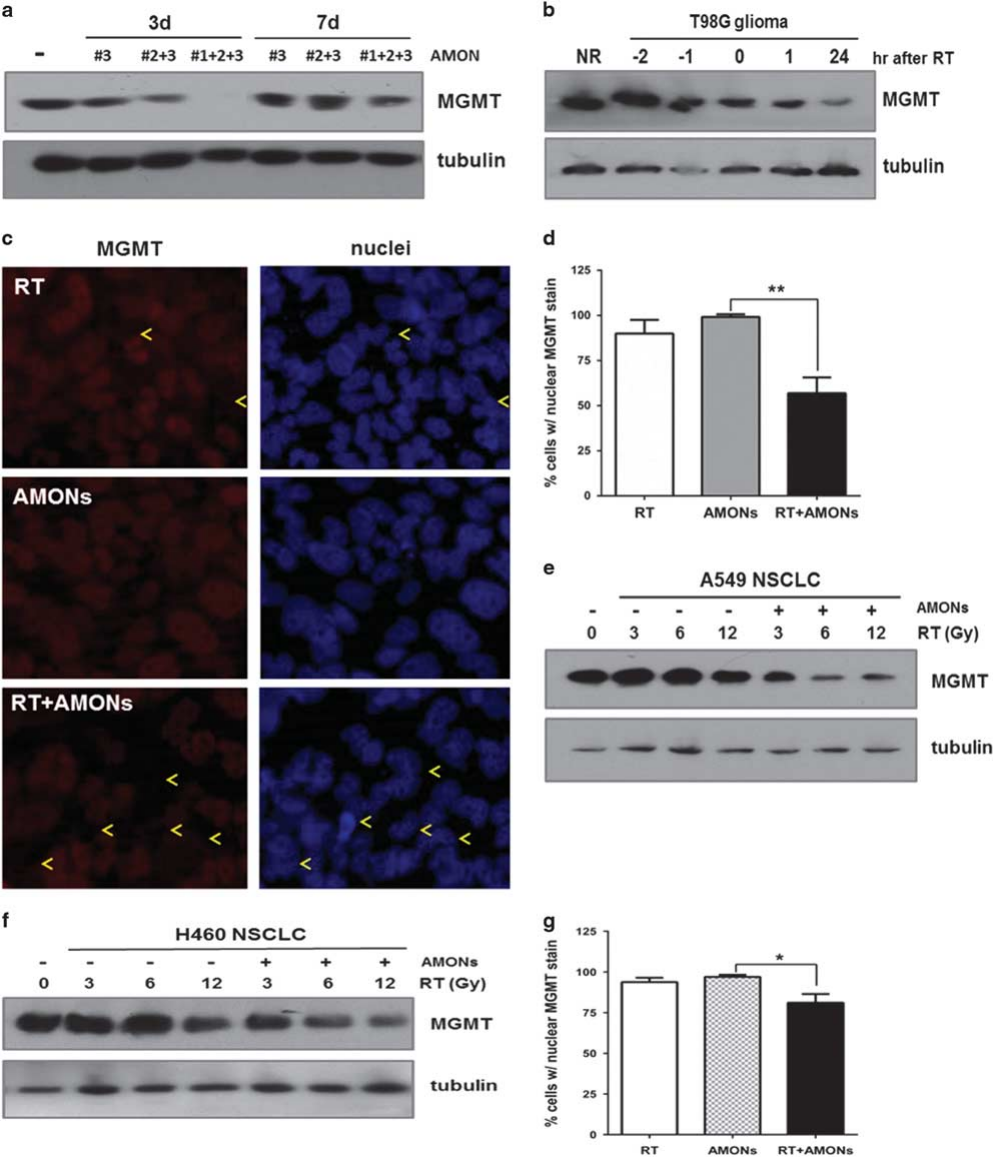
Radiation enhances anti-MGMT oligonucleotide (AMON) delivery and reduces O^6^-methylguanine DNA methyltransferase (MGMT) protein expression in human cancer cells. (**a**) Downregulation in MGMT expression after treatment with a single dose of AMONs (combination of sequences 1, 2 and 3 in 1:1:1 ratio; 5 μM each) in radiation-primed T98G cells was seen at 3 days, and MGMT expression returned to pretreatment baseline by 7 days; (**b**) Immunoblot demostrates the time-dependent downregulation of MGMT expression in T98G glioma cells treated with AMONs after a priming dose of non-ablative dose of radiation (1 Gy), compared with nonradiated (NR) cells. Maximal reduction in MGMT expression was noted after combination of sequences 1, 2 and 3 (1:1:1 ratio; 5 μM each were given 24 h after radiation); (**c**) Compared with cells treated with AMONs or radiation alone, radiation plus AMONs decreased the percentage of nuclear MGMT-positive-stained cells (indicated by arrowhead) in T98G cells; (**d**) Quantification of three random MGMT immunohistochemical fields shown in panel (**c**). Data are presented as mean±s.e.m.; (**e**, **f**) The dose of radiation required for optimal delivery of AMONs is tumor cell-type specific. Downregulation of MGMT by AMONs 3 days after AMONs in (**e**) A549 and (**f**) H460 non-small cell lung carcinoma (NSCLC) cells was in a radiation dose-dependent manner. RT, radiotherapy. (**g**) Quantification of 3 random MGMT IHC fields of H460 cells treated with RT, AMONs or RT+AMONs.

Expression of MGMT was verified in multiple cancer cell types and three MGMT-expressing solid tumor cell lines (T98G glioma, and H460 and A549 NSCLC) were selected for further experiments. Three AMON sequences were tested to identify to be the best combination to facilitate MGMT silencing (Table 1). T98G cells were exposed to sequences 2, 3 or the combination of 1, 2 and 3, 1 h after exposure to 1 Gy of ionizing radiation. Western blottings performed on cell lysates obtained 3 days after exposure to the sequences demonstrate almost complete (100%) silencing with the combination of sequences 1, 2 and 3, (Figure 1a) compared with sequences 2 and 3 or sequence 3 alone. The knockdown achieved with radiation-enhanced delivery of AMONs was transient, with knockdown at 3 days and restoration of MGMT expression by 7 days (Figure 1a). The combination of sequences 1, 2 and 3 (1:1:1 ratio) were used in future experiments in the current study unless specifically indicated. To determine the optimal timing for exposure to AMON with respect to exposure to radiation, T98G cells were exposed to AMONs at different time points, 2 h and 1 h before radiation as well as 1 and 24 h after radiation exposure. Of the 5 different time points we investigated, the best MGMT knockdown (87.4±5.3%) was achieved when cells were exposed to AMON 24 h after radiation compared with non-radiated control (Figure 1b). No time points >24 h after radiation were assessed. MGMT gene expression knockdown was confirmed with immunohistochemistry for MGMT protein (Figures 1c and d), which demonstrated a 43.2±6.5% reduction in nuclear MGMT staining in T98G cells exposed to AMONs 24 h after 1 Gy radiation. No significant reduction in nuclear MGMT was noted when cells were exposed to either radiation alone or when treated with AMON without radiation. The radiation dose needed to facilitate cellular delivery of AMONs varied between tumor cell lines. Knockdown could be achieved in T98G cell lines at 1 Gy while H460 and A549 cell lines required exposure to 6–12 Gy for best results with no-to-lesser degree of MGMT knockdown at lower doses of ionizing radiation (Figures 1a, e and f). Similar trends (18.2±4.2% reduction in nuclear MGMT staining) were found in H460 cells exposed to AMONS 24 h after 6 Gy radiation (Figure 1g). Alternate delivery techniques including scrape delivery and liposomal transfection agents, two well-described methods for *in vitro* delivery of oligonucleotides into the cytosol of cultured cells, were additionally tested (Supplementary Figure 1).^26^ A combination of sequences 1, 2 and 3 (1:1:1) at a dose of 15 μM resulted in 95±7.5% MGMT silencing at day 3 with scrape loading (Supplementary Figures 1a and b) or liposomal transfection agent (47.3±8.5% knockdown; Supplementary Figure 1c) techniques; MGMT silencing was not achieved when cells were exposed to AMONs without radiation priming, scrape loading or liposomal transfection agents (controls).

### Silencing MGMT using AMONs enhances the cytotoxicity of temozolomide

**Figure 2:**
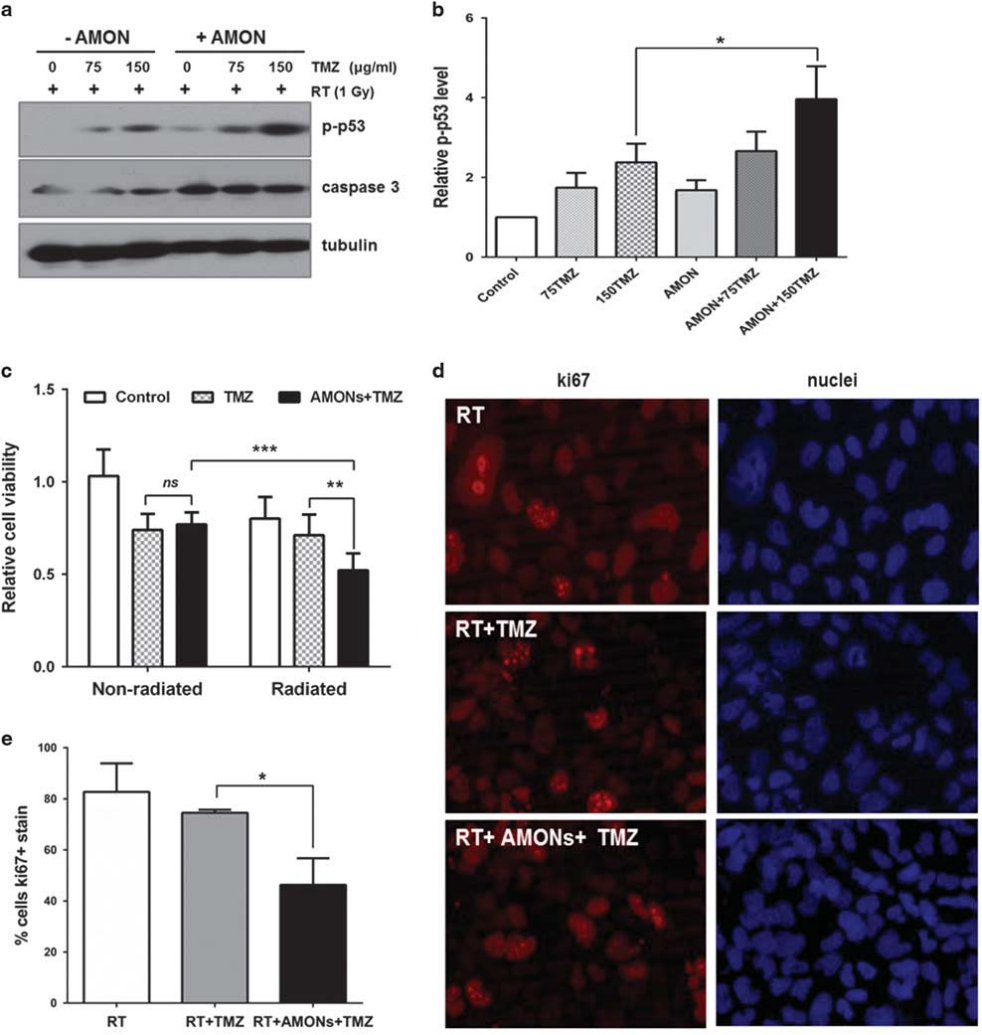
Downregulation of O^6^-methylguanine DNA methyltransferase (MGMT) by anti-MGMT oligonucleotides (AMONs) enhanced the *in vitro* cytotoxicity of temozolomide in T98G glioma cell lines. T98G cells were primed with 1 Gy followed 24 h later by AMONs (sequences 1, 2 and 3 in 1:1:1 ratio; 5 μM each). Two different doses of temozolomide (TMZ; 75 and 150 μg ml^−1^) in panels (**a** and **b**) and or 150 μg ml^−1^ temozolomide in pnels (**c**–**e**) was added at 2 days after AMONs. Untreated controls (radiation alone and radiation plus AMONs) were maintained. Whole-cell lysate was collected and subjected to western blotting analysis and cell viability assay was performed 1 day after temozolomide; (**a**) Enhanced cytotoxicity was noted in cells exposed to AMONs and temozolomide in a dose-dependent manner compared with controls. Early apoptosis of cell was indicated by phospho-p53 protein level; beta-tubulin was used as a loading control. (**b**) Semiquantification of western signal of phospho-p53 protein level in panel (**a**) of three seperately independent experiments; (**c**) Similar results were found when we use WST-1 cell viability assay. (**d**) Compared with radiation alone or radiation+TMZ, the adding of AMONs significantly decreased the percentage of ki67-positive-stained cells, suggesting that the cells were non-proliferating and cell cycle arrest at G_0_ stage. (**e**) Quantification of three random MGMT immunohistochemical fields shown in panel (**d**). Data are presented as mean±s.e.m. NS, not significant; RT, radiotherapy. Statistical significance indicated by *P<0.05. **P<0.01, ***P<0.001.

Once radiation-enhanced delivery of AMONs and MGMT silencing was confirmed, we tested whether this silencing could enhance the cytotoxicity of temozolomide. T98G cells were exposed to 1 Gy radiation (day 0), followed by AMONs (total of 15 μM; sequences 1, 2 and 3 in 1:1:1 ratio) on day 1 (Figure 1a). Cells were then exposed to 0, 75 or 150 μg ml^−1^ of temozolomide solution on day 4, respectively. Lysates were collected on day 5 for immunoblotting assay. Our results suggest that cells that received AMONs followed by temozolomide showed a dose-dependent increase in early apoptosis (phosphorylated-p53 protein) at day 1 compared with those that received no AMONs (Figures 2a and b). To confirm our findings, we performed WST-1 cell viability assay in T98G and H460 cells. Cells received 1 Gy of radiation on day 0 followed by either 150 μg ml^−1^ of temozolomide solution on day 3 or a combination of AMONs and temozolomide (AMONs on day 1 followed by temozolomide on day 3). Cells that received 1 Gy radiation only with no addition treatments served as controls. Cell viability was assessed on days 4–7. Our results show a statistically significant decline in T98G cell viability in the group that received AMONs and temozolomide compared those that received temozolomide only (Figure 2c) and a similar trend in H460 NSCLC cells. Immunohistochemistry (Figures 2d and e) demonstrates a statistically significant (*P*<0.01) decrease in the number of proliferating T98G cells (46.3±10.6% ki67+ cells) exposed to AMON and temozolomide compared with those that received temozolomide 24 h after 1 Gy radiation (74.5±1.2%) or radiation-only controls (82.7±11.2%).

### Radiation-guided enhanced delivery of IV administered AMONs downregulate MGMT in a subcutaneous tumor model

**Figure 3:**
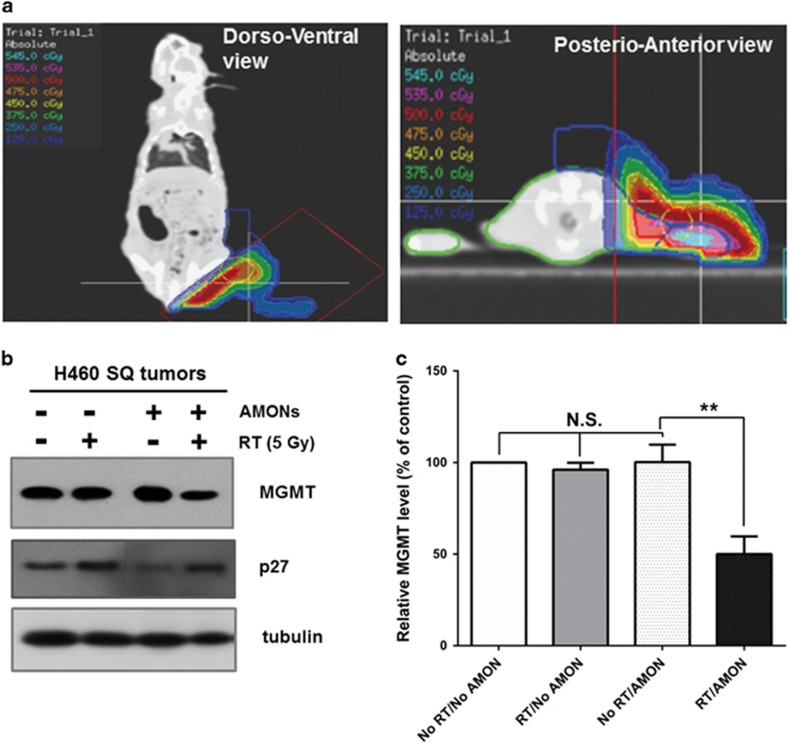
Radiation-guided enhanced delivery anti-MGMT oligonucleotides (AMONs) to reduce O^6^-methylguanine DNA methyltransferase (MGMT) expression in a H460 subcutaneous tumor model. Athymic nude rats were inoculated with H460 non-small cell lung carcinoma cells subcutaneously in both hind limbs (*n*=6); right limb tumors received a single dose of 5 Gy radiation and left ones served as non-radiated controls. AMONs (15 μg kg^−1^; intravenous) were administered 24 h after radiation to half (*n*=3) of animals and tumors were harvested 3 days later. (**a**) Representative CT scans with radiation field showing local delivery of radiation into right subcutaneous hindlimb tumors only; dosimetry confirmed that no radiation was delivered tumors on the contralateral hindlimb. (**b**) Immunoblot of MGMT and p27 of rat subcutaneous tumors; reduction in MGMT expression was found only in tumors that received both radiation and AMONs. MGMT expression was unchanged in those tumors that received AMONs or radiation only; upregulation of p27 observed in radiated (right) tumors. (**c**) Semiquantification of MGMT immunobloting signals from three independent experiments. NS, not significant; RT, radiotherapy.

Owing to inconsistent growth of T98G cells *in vivo*, MGMT-expressing H460 NSCLC subcutaneous tumors were used for our *in vivo* animal model. Six animals were subcutaneously injected with 2.5 × 10^7^ H460 cells in each of their hind limbs. When the tumors reached 10 mm in diameter (1 week after implantation), a single dose of 5 Gy of ionizing radiation was delivered only to the right hind limb tumor of each animal (Figure 3a). Half of the animals (*n*=3) then received IV AMONs 1 day after radiation, and tumors were harvested 3 days after exposure to AMONs. We found a 50±9.7% reduction in MGMT expression by western blotting assay in tumors primed with radiation prior to IV administration of AMONs. MGMT knockdown was not seen in control tumors that either received IV AMONs without prior radiation or received radiation alone (Figures 3b and c). Dosimetry confirmed radiation dose to each tumor site. Further, elevated p27 expression was used as a biomarker to confirm radiation exposure to the right hind limb tumors (Figures 3a and b).^34, 35^ We demonstrate the enhanced delivery and target downregulation only at tumor sites primed with a non-ablative dose of ionizing radiation. Pyrosequencing confirmed that the MGMT gene methylation status remained unchanged (Table 2), suggesting that the MGMT downregulation was due to posttranslational blocking rather than epigenetic silencing due to MGMT gene promoter methylation.

### Potential mechanism for radiation enhanced AMON delivery

**Figure 4:**
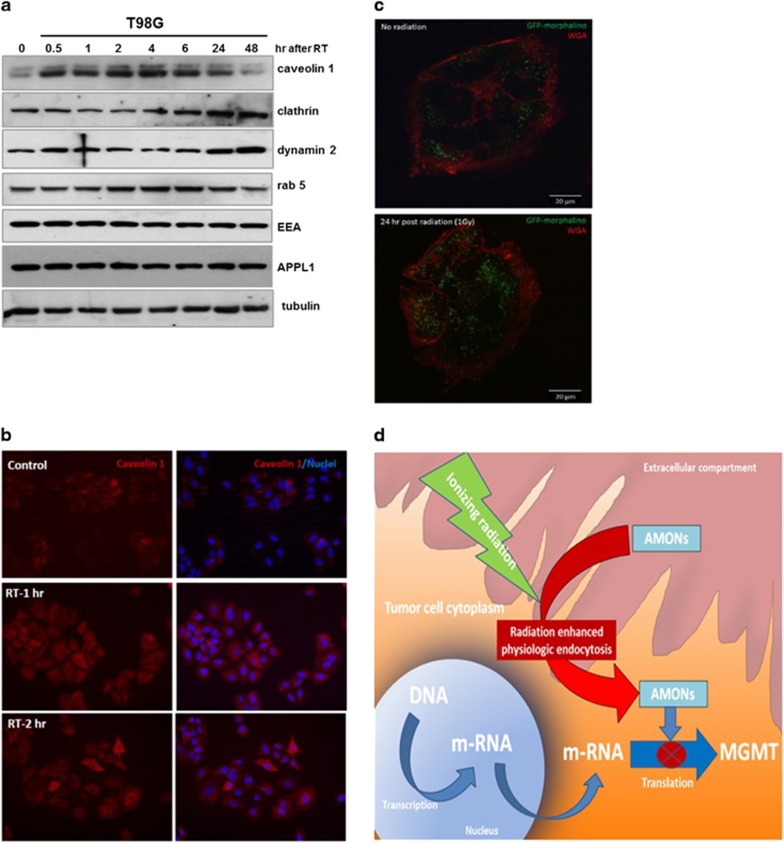
Radiation enhances the expression of key proteins in the physiological endocytosis pathway, possibly mediating intracellular delivery of morpholino into cancer cells. (**a**) Immunoblot of T98G cells showing that radiation affected the levels of endocytosis-related proteins caveolin-1, clathrin and dynamin 2, whereas others were unchanged (early endosome antigen (EEA), adaptor protein, phosphotyrosine interaction, PH domain and leucine zipper containing 1 (APPL1)); (**b**) Immunohistochemistry shown that radiation (6 Gy) affected caveolin-1 expression level and location in H460 non-small cell lung carcinoma; (**c**) Intracellular delivery of Green 3′-carboxyfluorescein morpholinos (1 μM; Gene Tool, LLC) in T98G cells 2 days after 1 Gy radiation. Red: wheat germ agglutinin (WGA) plasma membrane stain; (**d**) Scheme and principle of how anti-MGMT oligonucleotide (AMON) works to block O^6^-methylguanine DNA methyltransferase (MGMT) protein expression. rab, *Ras*-related protein; RT, radiotherapy.

To evaluate the mechanism by which radiation enhances AMON efficacy, the expression of key proteins involved in endocytosis pathway were assayed by western blotting from 0.5 to 48 h after radiation in T98G glioma cell line. Figure 4a demonstrates upregulation of caveolin 1 at 30 min after radiation, while clathrin and dynamin2 expression peaked at 24–48 h after ionizing radiation. This observation was confirmed in H460 NSCLC, and caveolin 1 protein expression was upregulated and translocated from plasma membrane to cytoplasmic and perinuclear locations by immunohistochemistry at 1 and 2 h after radiation (Figure 4b). Figure 4c demonstrates cellular uptake of non-specific fluorescent-tagged Morpholinos in T98G cells exposed to 1 Gy of radiation without the use of scrape delivery of transfection agents compared with morpholino treatment alone. These findings suggest that the radiation may enhance the physiological dynamin-mediated endocytosis and may have an important role in radiation-enhanced intracellular delivery of AMON (Figure 4d).

## Discussion

In this study, we describe the first use of a non-ablative priming dose of ionizing radiation to guide and enhance the delivery of AMONs to downregulate MGMT expression in human tumor cells, both *in vitro* and *in vivo* in a rat xenograft tumor model. MGMT expression is a critical mechanism for resistance to CRT in solid tumors, including glioblastoma, and decreasing MGMT expression would provide significant therapeutic benefit.^6, 12, 36, 37^ Various strategies to deplete MGMT have been previously reported and include the use of a dose-dense temozolomide regimen, pseudo-substrates of MGMT such as O^*6*^*-*Benzylguanine and O^*6*^-4-bromothenyl guanine, quinolone derivatives, S-adenosylmethionine and S-adenosylhomocysteine, and delivery of targeted short interfering RNA (siRNA) delivered in liposomes. Unfortunately, these methods face significant challenges in silencing efficacy, therapy delivery and toxic side effects. Our use of unmodified antisense morpholino oligomers for targeted protein silencing as well as the application of priming dose of non-ablative ionizing radiation to improved delivery overcomes many of these problems.

A morpholino oligomer backbone structure provides better sequence specificity than phosphorothioate-linked DNA (S-DNA) and siRNA due to (1) non-charged in physiological pH and hence do not bind electrostatically to proteins and are less likely to stimulate an immune response, limiting off-target effects, and (2) is resistant to the effects of nucleases.^38, 39^ Their stability in biological systems and target specificity makes them desirable for human therapeutics. The recent accelerated Federal Drug Administration approval of a modified morpholino oligomer-based exon-skipping therapy demonstrated a modest yet clinically relevant restoration of dystrophin in some patients with Duchenne muscular dystrophy, indicating morpholinos as a promising tool in the clinical context.^16, 39, 40^

Although, in theory, only a very small percentage of the antisense oligonucleotides administered are required to be taken up by cells to effectively modulate target proteins, target accessibility remains a major challenge (both delivery to the tumor tissue as well as delivery across the cell membrane in the tumor). Delivery has also been an issue in previous research studies that showed promising MGMT silencing in preclinical models, but the agents did not achieve clinically relevant intratumoral distribution even with direct intratumoral injection and convection-enhanced delivery techniques.^41, 42, 43, 44^ To facilitate the delivery of unmodified morpholinos across the cell membrane, the use of ‘scrape loading’, a vector, delivery reagents such as an endo-porter or structural modifications are required; however, these techniques have inherent limitations in the clinical setting. Structural improvements, such as the development of vivo-morpholinos that are morpholino oligonucleotides covalently linked, to an octa-guanidine dendrimer delivery moiety has significantly enhanced their intracellular delivery capabilities.^18^ However, these structural changes that increases their molecular weight may further limit their delivery across the BBB when administered systemically.^18^ Intracranial delivery is also limited by specific morpholino characteristics (that is, the structure, large size and polar nature of modified antisense oligonucleotides), temperature and concentration.^44, 45, 46, 47, 48, 49, 50^ The current study overcomes many of these challenges by using ionizing radiation to enhance delivery of MGMT targeting particles specifically to the tumor site. This highly targeted, site-specific delivery proves the added benefit of limiting the AMON-mediated silencing to only the ‘in-field’ site of radiation (that is, the tumor), thus decreasing toxic side effects of systemic MGMT inhibition, and being clinically applicable, as RT is standard of care in the treatment of glioblastoma.^1, 25^ We demonstrated that the use of non-ablative radiation enhances the delivery of unmodified morpholinos both *in vitro* and *in vivo*. A single dose of AMONs was able to transiently achieve near-complete MGMT protein knockdown *in vitro* and 50% knockdown *in vivo*. Our results are comparable to the degree of *in vitro* MGMT knockdown previously described with O^6^-BG and anti-MGMT siRNA encapsulated in cationic liposomes.^51, 52^ The dose of priming ionizing radiation needed for optimal delivery of AMONs appears to vary among different cancer cell types (Figures 1a, e and f). This may be due to inherent biological differences in sensitivity to radiation among various tumors.^53, 54, 55, 56^

Although there is emerging interest in the use of targeted drug delivery by use of external beam radiation and there have been reports of its use to enhance the delivery and activity of targeted agents such as nanoparticles and viral vectors, the mechanism involved in radiation-mediated enhanced delivery is unclear.^24, 42, 43, 57^ Owing to the time lag noted for optimal effect after radiation (Figure 1c), we hypothesize that upregulation of the physiological endocytic pathway may be responsible for the enhanced uptake of AMONs.^58, 59^ We found that dynamin is upregulated at 24–48 h after radiation while caveolin1 is upregulated within 30 min and stays upregulated for up to 24 h after radiation exposure. Treating cells with a cell-permeable inhibitor of dynamin, dynasore, did not reduce internalization of fluorescent-tagged non-specific morpholino (data not shown), suggesting the endocytic mechanism is dynamin independent.^60^ Ionizing radiation is known to upregulate multiple endocytosis pathways as well as increase surface antigens and receptors that could potentially facilitate endocytosis of AMONs.^24, 61^ The cell membrane lipid bilayer is naturally impermeable to complexes >1 kDa, alternate strategies involving the modulation of endocytosis using lipid-based drug delivery systems, nanoscale carriers and synthetic receptor targeting adapted for high affinity binding of targeting molecules and clathrin-dependent uptake are not novel and some of these are currently being evaluated in clinical trials.^62, 63, 64^ Radiation-mediated enhanced intracellular uptake as well as microbubble oscillation using ultrasound to deliver adeno-viral vectors, plasmid DNA or siRNA have been previously described by upregulation of the physiological endocytosis pathways and our study for the first time demonstrate the use of a priming dose of non-ablative ionizing radiation-enhanced delivery of unmodified morpholino oligomers to effectively knockdown target proteins.^24, 65, 66^

The major limitation in our study is the lack of an intracranial orthotropic MGMT-expressing glioblastoma brain tumor model. This limits our ability to determine the efficacy of AMONs to cross the BBB and silence MGMT. In this context, it should be noted that RT by itself is known to increase the BBB permeability and theoretically enhance the delivery of AMONs to the tumor site, from where radiation-enhanced upregulation of physiological endocytosis will facilitate its intracellular delivery.^67^ MGMT-expressing glioma cell lines are less invasive and are known to be difficult to grow in animal models.^68^ Further, the optimal combination or ratio of sequences was not determined. Although our study suggests that good cellular uptake was noted at 24 h after exposure of the radiation, the effects of radiation on uptake beyond the 24 h point is yet to be determined. As radiation is delivered in 1.8–2 Gy daily dose fractions over 6 weeks with daily temozolomide for patients with glioblastoma, a daily IV dosing schedule may be feasible to attain sustained MGMT silencing during this period.

Our findings provide preliminary evidence to suggest that upregulation of physiological endocytosis pathway may have an important role in the enhanced AMON uptake seen in tumor cells primed with a non-ablative dose of radiation (Figure 4). Further studies are being conducted to further elucidate the mechanism of morpholino uptake in radiation-primed cells. As previously described with other morpholino oligonucleotides, MGMT knockdown achieved with a single dose of AMONs using our approach was transient.^69^ In the context of glioblastoma therapy, this transient MGMT knockdown is ideal for timing therapy to achieve peak knockdown with the alkylating chemotherapy, thus limiting the potential risks associated with systemic and persistent suppression of this vital DNA repair mechanism.

A single dose of AMONs (10.5 mg kg^−1^; IV) in combination with 5 Gy local radiation achieved about 50% MGMT protein knockdown in our animal H460 subcutaneous tumor model, and this dose is comparable and scalable to the 30–50 mg kg^−1^ week^−1^ dose approved in treating Duchenne muscular dystrophy in clinical trial. As our goal was to determine whether targeted MGMT knockdown could be achieved, animals were killed and tumors were harvested at early time points. Significant differences in cytotoxicity were not expected at early time point, and the role of concurrent alkylating chemotherapy is an important goal for future studies. MGMT mediates chemoresistance and is frequently seen in various solid tumors, including non-small cell lung carcinoma, melanoma and breast carcinoma. Another hurdle for clinical translation of this approach to brain tumor therapy is the delivery across the BBB.^70, 71^ Additional experiments to determine the optimal mode of delivery across the BBB with MGMT-expressing brain tumors are currently underway using a vivo-morpholino backbone.^18^ Successful delivery across the BBB would be necessary to be effective in brain tumors, and we used ‘standard’ rather than vivo-morpholino backbone that has a larger molecular size for our sequences and are less likely to cross the BBB. Future experiments will evaluate additional augmented delivery techniques such as intra-arterial route with osmotic blood brain disruption, convection enhanced delivery and so on for delivery of AMONs across the BBB.

## Conclusion

This proof-of-concept study demonstrates that MGMT can be silenced in multiple cancer cell lines *in vitro* and in a subcutaneous tumor model with enhanced delivery of AMONs with a non-ablated dose radiation. Our findings suggest that upregulation of key endocytosis proteins may be responsible for the enhanced delivery after radiation. This new technique for targeted delivery of unmodified morpholino oligomers has the potential for direct translation. We used MGMT as the prototype to demonstrate the proof of concept for modulating a cancer-specific protein; this technique can potentially be used to target other proteins. As RT is widely used in the management of various cancers, targeted delivery of systemically administered AMONs only to the ‘field of radiation’ will limit the off-target side effects. Newer molecular targets and further improvement in morpholino structure are expected to further enhance stability and efficacy of morpholino-mediated protein silencing.

## Figures and Tables

**Table 1:** Summary of oligonucleotide sequences used in this paper

*Morpholinos*	*Nucleotide sequence 5′*→*3′*	*No. of bp*^a^	*MW*
*Anti-MGMT specific*			
Sequence #1	5′-TTTCGTGCAGACCCTGCTCTT-3′	21	7037
Sequence #2	5′-TTCCATAACACCTGTCTGGTT-3′	21	7045
Sequence #3	5′-ATTCCTTCACGGCCAGTCCTT-3′	21	7006

Nonspecific	5′-CCTCTTACCTCAGTTACAATTTATA-3′	25	8328
Fluorescent nonspecific	5′-CCTCTTACCTCAGTTACAATTTATA-carboxyfluorescein-3′	25	8817

Abbreviations: A, adenosine; C, cytosine; G, guanine; MGMT, O^6^-methylguanine DNA methyltransferase; MW, molecular weight; T, thymine.

a Number of base pair in nucleotides.

**Table 2:** Summary of MGMT DNA promoter methylation percentage of H460 subcutaneous tumors after treatment of radiation and AMONs

*Treatment*^a^	*MGMT DNA promoter methylation*^b^ *(%)*			
	*Sample#1*	*Sample#2*	*Sample#3*	*Mean±s.d.*
No RT/no AMONs	0	0	0	0±0
RT/no AMONs	1	1	0	0.67±0.58
No RT/AMONs	0	0	0	0±0
RT/AMONs	1	1	1	1±0

Abbreviations: AMON, anti-MGMT morpholino oligonucleotide; MGMT, O^6^-methylguanine DNA methyltransferase; RT, radiotherapy.

a RT (5 Gy) and AMONs (10.5 mg kg^−1^; intravenous).

b Percentage of methylation of MGMT DNA promoter was determined by pyrosequencing detailed in Materials and Methods section.
