# Supplementary material for: Enhancing the cytotoxicity of chemoradiation with radiation-guided delivery of anti-MGMT morpholino oligonucleotides in non-methylated solid tumors
Source: Cancer Gene Ther. 2017 Jul 28;24(8):348–57. doi: 10.1038/cgt.2017.27 (PMC5605678; doi:10.1038/cgt.2017.27)
Supplement: Supplementary Information [file cgt201727x1.docx]

**Supplemental Data:**

**Figure 1.** **Novel anti-MGMT oligonucleotides (AMON) can knockdown MGMT protein expression among different cancer cells. A)** MGMT protein knockdown was achieved by scrape delivery of AMON sequences in T98G cells. Whole cell lysates were collected and subjected to western blot analysis at 3 days after treating with either single AMON sequence 1, 2 or 3 (15 μM) or combination of sequences 1, 2 and 3 (1:1:1 ratio; 5 μM each). Best results were achieved with sequence 3 or the combination of sequences 1, 2 and 3; **B)** MGMT knockdown by AMONs is dose-dependent in H460 NSCLC cells compared to scrapeonly (lane 1) or non-specific oligonucleotide sequence (lane 2). **C)** MGMT knockdown by AMON via either scrape or DharmaFECT transfection agent delivery was transient in T98G cell line. The MGMT protein was reduced by AMON at 3 days and returned back to normal level at 7 days after delivery. TA: Transfection agent.

**Figure 2. Down-regulation of MGMT by AMONs enhanced the in vitro cytotoxicity of temozolomide in H460 NSCLC cells. H460 cells?** cells were primed with 6 Gy followed 24 hrs later by AMONs (sequences 1, 2 and 3 in 1:1:1 ratio; 5 μM each). Temozolomide (TMZ; 150 μg/mL) was added at 2 days after AMONs. Untreated controls (radiation alone and radiation plus AMONs ) were maintained. WST-1 cell viability assay was performed 1 day after temozolomide; **A)** Enhanced cytotoxicity was noted in cells exposed to AMONS and Temozolomide compared to controls; **B)** Quantification of 3 random MGMT IHC fields shown the adding of AMONs significantly decreased percentage of ki-67 positive stained cells compared to radiation alone; Data were presented as mean ± SD.
