# Supplementary figures and images for: Enhancing the cytotoxicity of chemoradiation with radiation-guided delivery of anti-MGMT morpholino oligonucleotides in non-methylated solid tumors
Source: Cancer Gene Ther. 2017 Jul 28;24(8):348–57. doi: 10.1038/cgt.2017.27 (PMC5605678; doi:10.1038/cgt.2017.27)

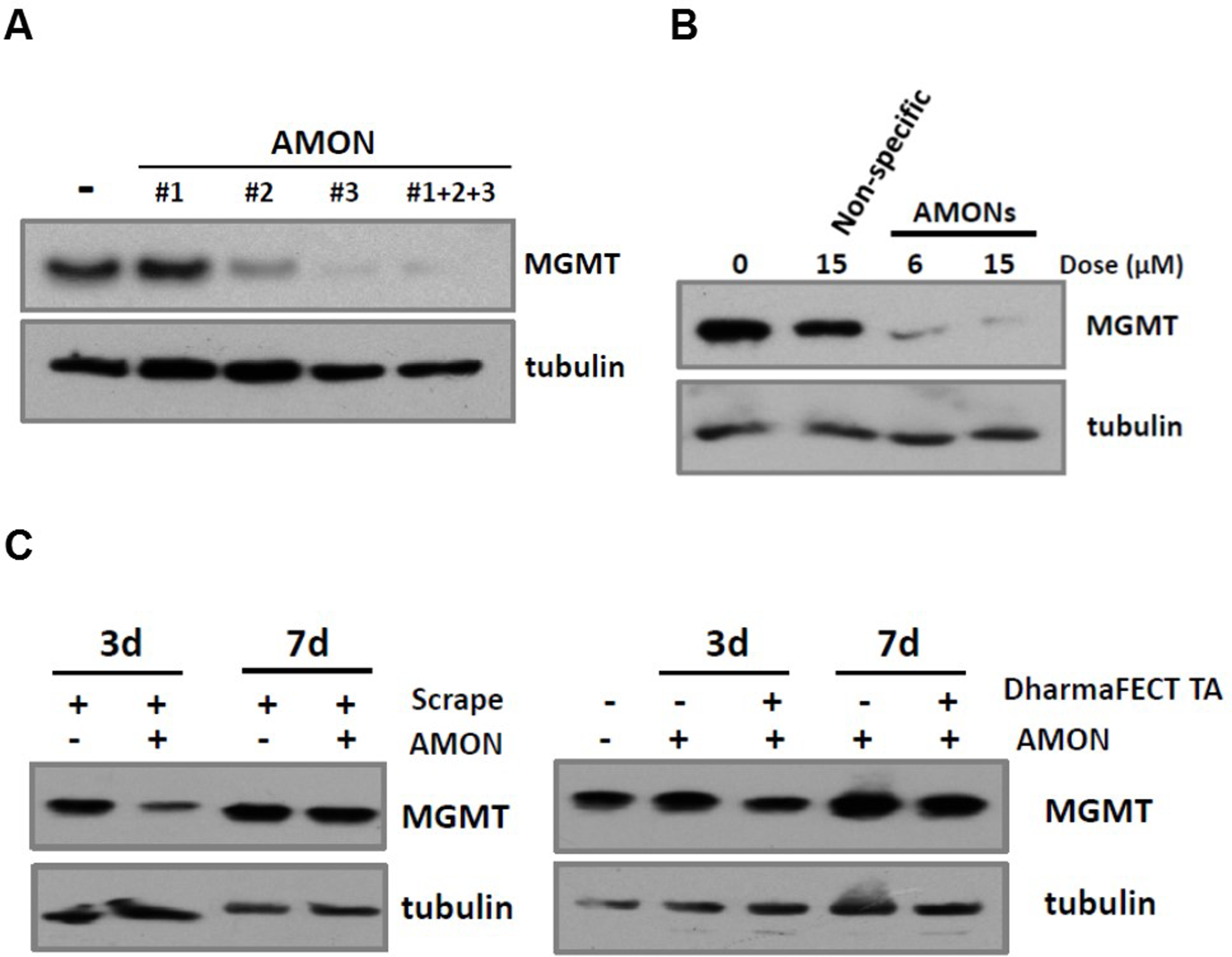

Supplement: Supplementary Figure 1 [file cgt201727x2.tif]

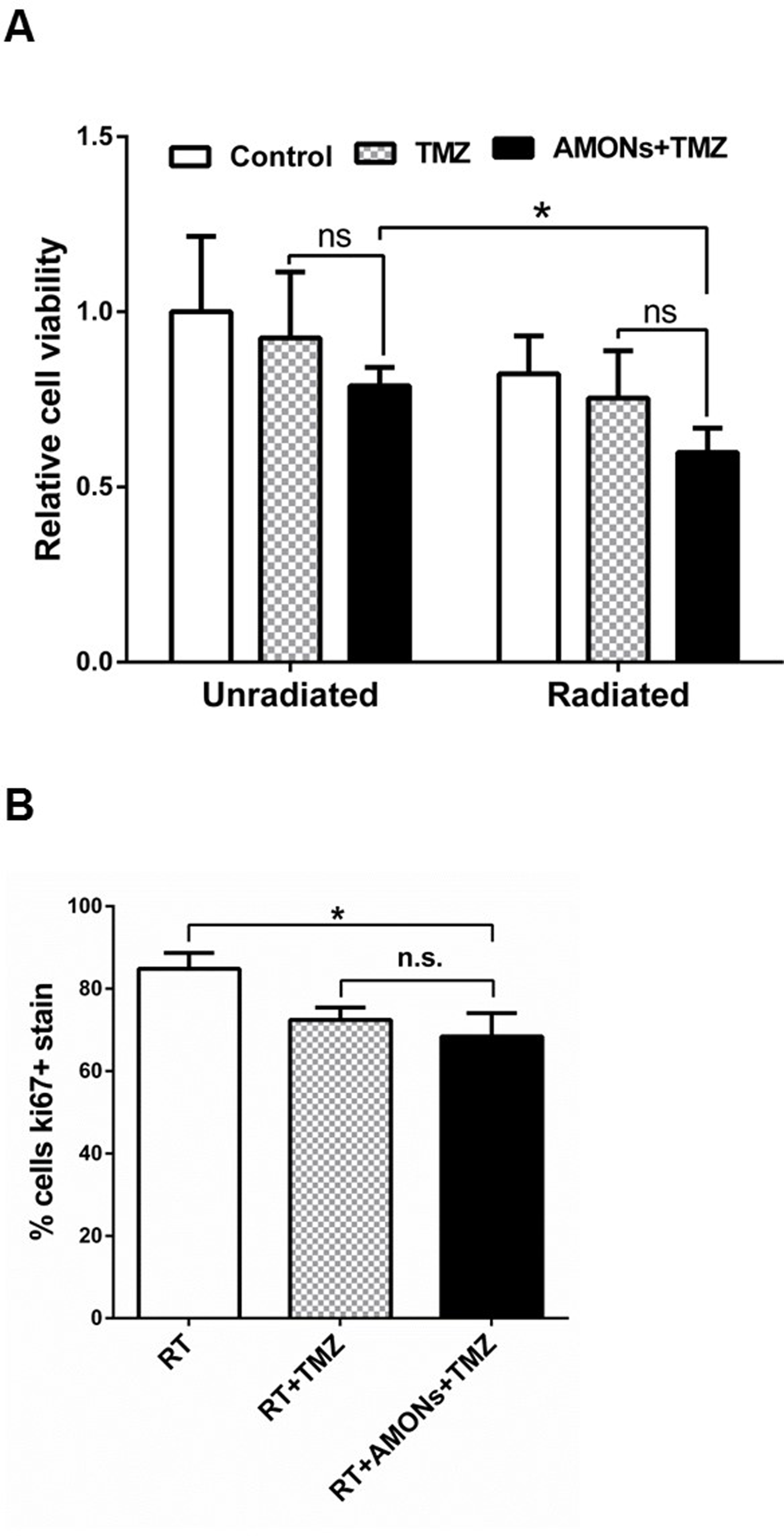

Supplement: Supplementary Figure 2 [file cgt201727x3.tif]
